# Divergent *Wnt8a* Gene Expression in Teleosts

**DOI:** 10.1371/journal.pone.0085303

**Published:** 2014-01-20

**Authors:** Nesrin Mwafi, Carlo A. Beretta, Alessio Paolini, Matthias Carl

**Affiliations:** Heidelberg University, Medical Faculty Mannheim, Department of Cell and Molecular Biology, Mannheim, Germany; Chang Gung University, Taiwan

## Abstract

The analysis of genes in evolutionarily distant but morphologically similar species is of major importance to unravel the changes in genomes over millions of years, which led to gene silencing and functional diversification. We report the analysis of *Wnt8a* gene expression in the medakafish and provide a detailed comparison to other vertebrates. In all teleosts analyzed there are two paralogous *Wnt8a* copies. These show largely overlapping expression in the early developing zebrafish embryo, an evolutionarily distant relative of medaka. In contrast to zebrafish, we find that both maternal and zygotic expression of particularly one *Wnt8a* paralog has diverged in medaka. While *Wnt8a1* expression is mostly conserved at early embryonic stages, the expression of *Wnt8a2* differs markedly. In addition, both genes are distinctly expressed during organogenesis unlike the zebrafish homologs, which may hint at the emergence of functional diversification of *Wnt8a* ligands during evolution.

## Introduction

Teleosts are particularly suitable for comparative studies to elucidate evolutionary events resulting in genomic diversity. With as many as 25.000 species arising within the last 250 million years, it is the most diverse group of vertebrates and with the increasing number of genome sequences available an invaluable resource for genome comparisons. Amongst the sequenced fish genomes are the zebrafish (*Danio rerio*) [1] and the medakafish (*Oryzias latipes*) [2], which share their last common ancestor about 110 million years ago [3]. Due to an ancient genome duplication, teleosts frequently have two copies of genes represented only once in the mammalian genome [4]–[6]. The Duplication-Degeneration-Complementation (DDC) model of gene evolution has helped framing ideas about how genomes and gene functions evolve following duplication events [7]. For instance, gene paralogs can lose their function during evolution or the coding sequence of one duplicate can change resulting in novel protein functions (neo-functionalisation). Past research has also shown that mutations in gene regulatory regions can result in the acquisition of a new site or time of expression and a paralog's function is changing (sub-functionalisation) [7]–[10]. Frequently, the expression domain and function of the single mammalian gene homolog is equivalent to the combination of the expression domains and functions of the two fish paralogs. This sub-functionalization of genes in fish can be used to dissect the whole range of gene functions of their non-duplicated mammalian homologs [11], [12]. Thus, it is of fundamental importance to study gene expression and function in related and morphologically similar but evolutionarily distant species to gain insights into the functional complexity of a given gene and to address questions regarding gene/genome evolution also compared to mammalian systems.

The Wnt signaling pathway is highly conserved and amongst the most intriguing signaling cascades studied to date. Its exceptionally wide range of functional properties ranging from cell proliferation and tissue homeostasis to cell differentiation and cellular diversity in development and disease has inspired many researchers investigating Wnt signaling [13], [14]. However, by today, 28 different Wnt ligands have been identified in zebrafish [15] and only a very small subset of ligands has been analyzed in medaka [16]. *Wnt8* genes are amongst the most prominent ligands in Wnt signaling. In vertebrates, two *Wnt8* genes are present in the genome, *Wnt8a* and *Wnt8b*. In teleosts, the genome duplication resulted in the generation of an additional *Wnt8a* paralog in close genomic proximity and in tandem to the first [17]. This arrangement appears very conserved amongst teleosts [17]–[19]. Interestingly, besides a transcript for the second *Wnt8a* paralog, a bicistronic *Wnt8a* transcript encoding both Wnt8a proteins has been identified in zebrafish [17].

The two *Wnt8a* paralogs are largely overlappingly expressed during early zebrafish development and appear to exert similar but distinct functions [17], [18]. However, it is not known whether these features are conserved within the teleost lineage. Moreover, an expression or functional analysis at later stages has not been carried out.

Here we report the expression analysis of the two medaka *Wnt8a* paralogs during embryonic development. Like for other teleosts, the two genes are arranged in close proximity to each other in the genome. However, unlike in zebrafish, both maternal and zygotic expression of the medaka *Wnt8a* genes differs in various developing tissues. Our data indicate that *Wnt8a* gene expression has diverged between distantly related teleost species and implies that they may have acquired different functions during evolution. Moreover, we find sites of medaka *Wnt8a* expression during organogenesis stages, which we did not detect in zebrafish but are found also in mammals with the exception of the caudal hematopoietic system and gall bladder.

## Materials and Methods

### Ethics statement

The approval for all animal work carried out was obtained from the Regierungspräsidium Karlsruhe (35-9185.64).

### Fish maintenance

Wildtype *Oryzias latipes* from a closed stock at the University Heidelberg and the *Danio rerio* AB x TL line were kept as described [20]–[22].

### Whole mount in situ labeling

Whole mount in situ hybridization using digoxigenin labeled RNA riboprobes for medaka *Wnt8a1* and *Wnt8a2* and zebrafish *Wnt8a ORF1* and *Wnt8a ORF2* were perform as described [23], [24].

### Cloning of the full length medaka *Wnt8a1* and *Wnt8a2* genes

Total RNA was isolated from 1, 2 and 3 dpf medaka embryos using Trizol® (Invitrogen, Darmstadt, Germany). First strand cDNA was prepared according to the manufacturer's protocol (Invitrogen SuperScript®III First-Strand kit, Darmstadt, Germany). To clone both medaka full-length *Wnt8a* genes from the synthesized cDNA, PCR was performed using the Taq DNA polymerase kit (QIAGEN, Hilden, Germany) with the following primers.

Wnt8a1 forward 5′ AGCGTGGAGGGAGGCTGCAT 3′, Wnt8a1 reverse 5′ TGGAGTGCCCCGTGTTCTGT 3′, Wnt8a2 forward.

5′ AGGAAAATTGAAGAAGCGAACCAGGA 3′ and Wnt8a2 reverse.

5′ AGCCGTAATCTTTCATCTGGGGGC 3′. PCR program: Denaturation for 30 sec at 95°C, annealing for 30 sec at 70°C for *Wnt8a1* and 62°C for *Wnt8a2* followed by extension for 1 min at 72°C for a total of 35 cycles. The first cycle was preceded with initial denaturation for 3 min at 95°C and the last cycle was followed by additional extension for 3 min at 72°C and cooling at 25°C for 30 sec. The purified full-length *Wnt8a* cDNAs (*Wnt8a1*: 1349 bp and *Wnt8a2*: 1110 bp) were cloned into the pCRII-TOPO vector (Invitrogen, Darmstadt, Germany) according to the manufacturer's instructions and sequenced.

### RT-PCR detection of *Wnt8a* transcripts in medaka and zebrafish

To detect maternal and zygotic expression of medaka *Wnt8a* genes, we isolated total RNA from medaka embryos at 2-cells, 4-cells, 16-cells and 40% epiboly. The synthesized first strand cDNAs were used as templates for PCR carried out as above to obtain the *Wnt8a2* cDNA. To obtain a 530 bp long *Wnt8a1* fragment, we used the same PCR conditions as above with an annealing temperature of 60°C and for Wnt8 the following primers: Wnt8a1 forward 5′ AGCGTGCAAGTGTCACGGCG 3′, Wnt8a1 reverse 5′ CACGGTCCCTGCGCTTCGTT 3′.

Total RNA from zebrafish was isolated from embryos at 80% epiboly and from heads dissected posterior to the hindbrain and the remaining tails at 48 hpf. To obtain a 217 bp long *Wnt8a ORF1* cDNA fragment and a 264 bp long *Wnt8a ORF2* fragment (see supporting information Figure S1I), the PCR conditions were used as above with an annealing temperature of 62°C with the following primers: Wnt8a ORF1 forward 5′ GCTGTCAAAGCAACGCTCAA 3′, Wnt8a ORF1 reverse 5′ AGCTGAACGTGTCCGCTATT 3′, Wnt8a ORF2 forward 5′ GGATGTAGCGACAACGTGGA 3′, Wnt8a ORF2 reverse 5′ GAGTTTCTGGGCCTGATCGT 3′.

### Phylogenetic analysis

The genomic browsers: Ensembl (http://www.ensembl.org/index.html) and NCBI (http://www.ncbi.nlm.nih.gov/) were used to collect the sequences of *Wnt8a* transcripts: *Homo sapiens* (Wnt8a: ENST00000398754); *Pan troglodytes* (Wnt8a: ENSPTRT00000031975); *Bos taurus* (Wnt8a: NM_001192370.1); *Equus caballus* (Wnt8a: ENSECAT00000015524); *Mus musculus* (Wnt8a: ENSMUST00000012426); *Rattus norvegicus* (Wnt8a: ENSRNOT00000064574); *Ciona intestinalis* (WntA202: ENSCINT00000012046); *Xenopus tropicalis* (Wnt8a: ENSXETT00000008323); *Xenopus laevis* (Wnt8a: NM_001088168.1); *Taeniopygia guttata* (Wnt8a: ENSTGUT00000001220); *Tetraodon nigroviridis* (Wnt8a-203: ENSTNIT00000002526, Wnt8a-202: ENSTNIT00000001194); *Gallus gallus* (Wnt8C: ENSGALT00000009817); *Oryzias latipes* (Wnt8a1 (formerly named WNT8A (1 of 2): ENSORLT00000009606, Wnt8a2 (formerly named WNT8A (2 of 2): ENSORLT00000009585); *Gasterosteus aculeatus* (Wnt8a_1.2: ENSGACT00000024478, Wnt8a_2.2: ENSGACT00000024475); *Danio rerio* (Wnt8a ORF1: ENSDART00000017635, Wnt8a ORF2: ENSDART00000105649); *Takifugu rubripes* (Wnt8.1: AY628150, Wnt8.2: AY628150). At NCBI, both *Takifugu rubripes Wnt8* genes share one accession number and are named wnt8 bicistronic mRNA with nucleotides 1-1092 bp coding for the Wnt8.1 protein and 1552–2604 bp coding for the Wnt8.2 protein.

The *Wnt8a* cDNA sequence alignments and the phylogenetic trees were built using Geneious software. The sequences were aligned using the global multiple alignment method with free end gaps. The tree was built using the Phylogenetic Maximum Likelihood method (PhML) [25] and the Hasegaw-Kishino-Yano (HKY85) substitution model with 10.000 bootstrap replicates using *Ciona intestinalis* as outgroup. To disentangle nodes in the tree, MrBayes analysis was applied [26]. The MCMC (Markov chain Monte Carlo) was used to calculate the posterior probabilities of phylogenetic trees.

## Results and Discussion

### *Wnt8a* ligands are conserved amongst vertebrates

**Figure 1:**
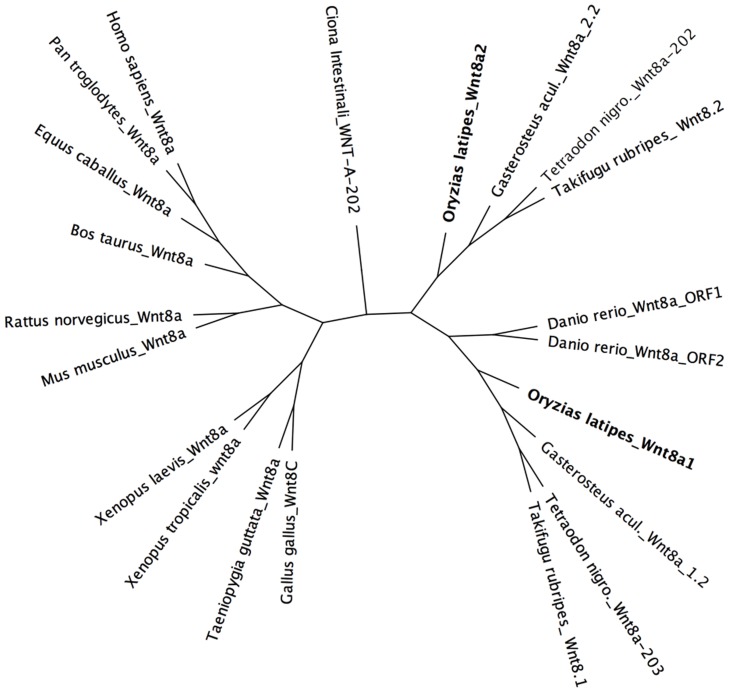
Phylogenetic analysis of *Wnt8a* genes. The medaka genome contains two *Wnt8a* genes (bold). The *Wnt8a* cDNA sequence alignment of various species shows that the medaka paralogous copies cluster well with those of evolutionarily related teleosts. acul., aculeatus; nigro., nigroviridis.

Like in other teleosts [17]–[19], the two medaka *Wnt8a* paralogs are organized in a tandem arrangement in the genome. We isolated the full length coding sequences of medaka *Wnt8a1* (formerly named *Wnt8-like*) [16] and *Wnt8a2*. Phylogenetic analysis shows that the medaka *Wnt8a* paralogs cluster well with the respective *Wnt8a* paralogs of the evolutionarily close Fugu and Tetraodon (Figure 1). In contrast, the zebrafish *Wnt8a* genes cluster together on the *Wnt8a1* branch of the tree. Consistent with its evolutionary distance, the medaka Wnt8a1 protein shares higher homology with its Fugu homolog (78% identical amino acids (aa)) rather than with zebrafish Wnt8a ORF1 (also named Wnt8.1, Wnt8a or Wnt8a.1) (69% identical aa). However, the second Wnt8a protein, Wnt8a2, appears similarly conserved (Fugu Wnt8.2 shares 71% identical aa with medaka Wnt8a2 and 72% with zebrafish Wnt8a ORF2; also named Wnt8.2 [17]).

Like zebrafish Wnt8a ORF1 (63% identical aa compared to 62% of Wnt8a ORF2), the medaka Wnt8a1 protein is most similar to the mammalian single Wnt8a protein (60% identical aa compared to 56% of Wnt8a2).

This indicates that the medaka genome contains two paralogous *Wnt8a* copies, which are well conserved within closely related teleosts and that *Wnt8a1* is the gene more similar to the single mammalian *Wnt8a*.

### Differential initiation of medaka *Wnt8a* gene paralog expression

Zebrafish *Wnt8a ORF1* is expressed maternally [27] and the earliest *Wnt8a* expression in other animal models has been reported in the posterior epiblast prior to gastrulation in mice and chick [28], [29]. Early zygotic zebrafish *Wnt8a* expression is found in lateral and ventral marginal cells of the blastoderm [17], [27] (Figure S1A and B). Its expression in the embryonic shield remains controversial. This gene was initially described to be expressed in the shield and downregulated subsequently [27]. A later study mentioned that *Wnt8a ORF1* (and *Wnt8a ORF2*) is not expressed in the shield until about 75% epiboly [17]. At subsequent stages, *Wnt8a ORF1* is expressed in the paraxial mesoderm [27] and *Wnt8a ORF2* remains expressed in marginal cells similar to its paralog [17].

**Figure 2:**
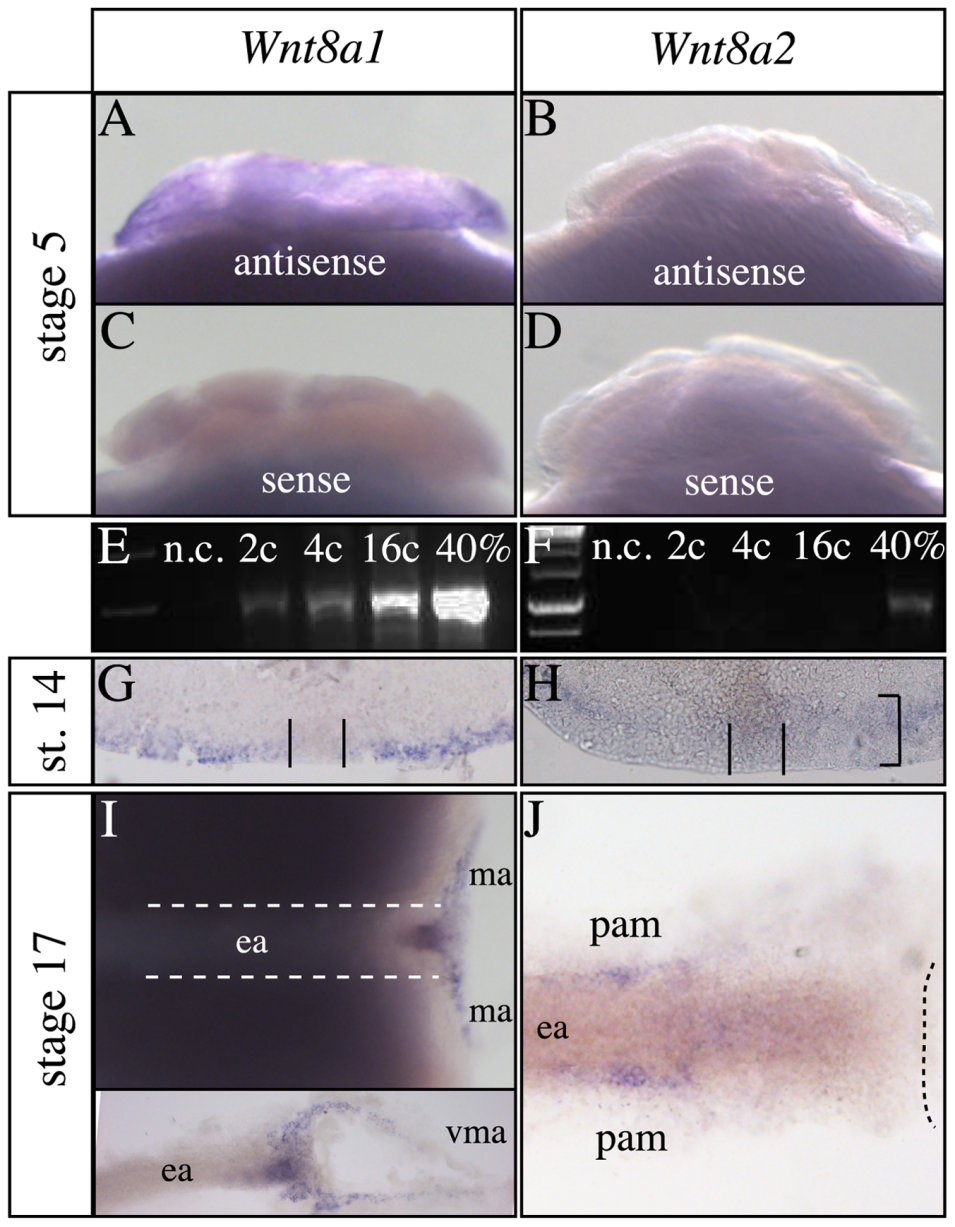
Differential early *Wnt8a1* and *Wnt8a2* gene expression. (A–D) 8-cell stage embryos are shown with the animal pole to the top. Sense RNA probes were used to validate the maternal *Wnt8a1* expression. (E,F) RT-PCR for *Wnt8a1* and *Wnt8a2* transcripts on extracted RNA at stages indicated to the left. (G,H) Pictures focused on the dorsal margin of the dissected blastoderm of embryos at 40% epiboly with animal pole to the top. While *Wnt8a1* (G) is expressed in cells of the blastoderm margin but not in cells of the shield (framed by black lines), *Wnt8a2* (H) is weakly expressed in cells of the shield and the margin. Open rectangle indicates the broad *Wnt8a2* expression domain at the margin. (I,J) Embryos at 80% epiboly with anterior to the left; (I) dotted lines frame the developing embryonic axis; inset and (J) show embryos dissected from the yolk for clarity. (J) Dotted line marks the blastoderm margin. 40%, 40% epiboly; c, cells; ma, margin; ea, embryonic axis; n.c., negative control; pam, paraxial mesoderm; st., stage; vma, ventral margin.

We find that medaka *Wnt8a1* is maternally expressed (Figure 2A,C,E), while we did not detect any *Wnt8a2* transcripts by whole mount in situ hybridization or RT-PCR (Figure 2B,D,F). The early zygotic medaka *Wnt8a1* expression is well conserved amongst vertebrates and is seen in lateral and ventral marginal cells at 40% epiboly with the exception of the shield (Figure 2G) [16], [17], [27], [28], [30]–[32]. At this stage, *Wnt8a2* is expressed weakly in a slightly broader region of the blastoderm margin including cells of the shield (Figure 2H). At late gastrulation, medaka *Wnt8a1* is also found in cells of the dorsal blastoderm margin with lower expression in the ventral part similar to its homologs in other vertebrates [17] (Figure 2I and inset). However, no *Wnt8a2* transcripts are found in any of the marginal cells. Therefore the temporal expression of medaka *Wnt8a2* in the shield and the blastoderm margin markedly differs from medaka *Wnt8a1* and zebrafish *Wnt8a ORF2*. This may imply functional differences of the medaka *Wnt8a* genes during early embryonic development, which may be more prominent than in zebrafish [17].

Shortly before the end of gastrulation, we find *Wnt8a2* transcripts specifically in the paraxial mesoderm (Figure 2J). This transient *Wnt8a2* expression is similar to the reported expression of zebrafish *Wnt8a ORF1* and the single *Wnt8a* genes of Xenopus and mouse [17], [28], [33], [34] (Table 1).

**Table 1:** Sites of *Wnt8a* gene expression in medaka, zebrafish, Xenopus and mouse.

	Medaka *Wnt8a1* *Wnt8a2*	Zebrafish *Wnt8.1 Wnt8a.2*	Xenopus *Wnt8a*	Mouse *Wnt8a*
maternal	+ −	+ n.r.	n.r.	−
blastoderm margin/embryonic ectoderm	+ +*	+ +	+	+
paraxial mesoderm	− +	+ n.r.	+	+
tailbud	+ +	+ +	+	+
cht	− +	− −	n.r.	n.r.
gut/oesophagus	+ −	− −	+**	+**
gall bladder	+ −	− −	n.r.	n.r.
heart	+ −	− −	n.r.	+
swim bladder	+ −	− −	n.a.	n.a.
otic vesicles	− +	− −	n.r.	+
brain	− −	− +	+	+
fins/limbs, branchial arches, eye	− −	− −	n.r.	+
main references	(Yokoi et al., 2003) this report	(Kelly et al., 1995) (Lekven et al., 2001) (Narayanan et al., 2011) this report	(Smith and Harland, 1991) (Christian et al., 1991) (Christian et al., 1993) (In der Rieden et al., 2010)	(Bouillet et al., 1996) (Jaspard et al., 2000) (Summerhurst et al., 2008) (Martin et al., 2012)

*Wnt8.1* and *Wnt8.2* correspond to zebrafish *Wnt8a ORF1* and *Wnt8a ORF2*, respectively.

* , very transient; **, hindgut; +, expressed by in situ hybridisation; −, not expressed by in situ hybridisation. Abbreviations: cht, chaudal haematopoietic tissue; n.a., not applicable; n.r., not reported.

### Medaka *Wnt8a* expression during somitogenesis and organogenesis

During zebrafish somitogenesis, both *Wnt8a* genes are expressed in cells of the tailbud [17], [35] (Figure S1C and D). At 2 and 4 days of development, we find ubiquitous staining for both genes in the developing brain and no staining in the trunk and tail (Figure S1E–H). However, the same holds true for the genes' sense probes suggesting that the antisense probes give rise to background color. Indeed, using RT-PCR on RNA extracted from dissected heads and tails of 2 dpf embryos, we observed low amounts of cDNA products for both genes in both regions (Figure S1I). This suggests that also during stages following somitogenesis both zebrafish *Wnt8a* genes are expressed at low levels, which, however, is not detectable by in situ hybridization. In mouse and chick embryos *Wnt8a* expression is confined to the fore- and midbrain as well as limbs and branchial arches [36]. Furthermore, expression in the gut, heart and otic vesicles has been reported for mouse embryos [36]–[38]. Thus, many of the later *Wnt8a* expression domains may not be conserved between zebrafish and mouse and chick (Table 1).

**Figure 3:**
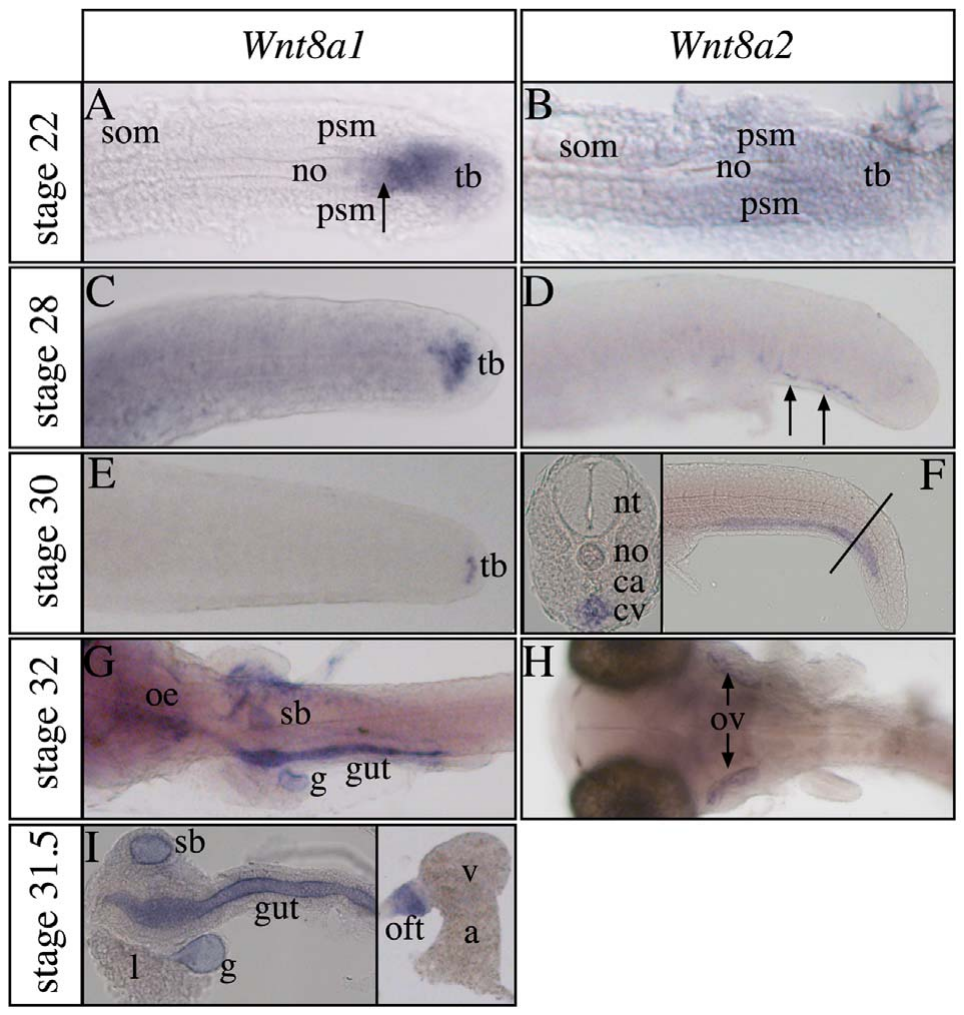
*Wnt8a* gene paralog expression pattern differ during somitogenesis and organogenesis. (A,B,E,G–I) Dorsal and (C,D,F) lateral views with anterior to the left, (A–F) focused on the tail region. (A,B) At 38 hours post fertilization (hpf), *Wnt8a1* transcripts are found in the axial part of the tailbud including the posterior end of the notochord (arrow), while *Wnt8a2* is widely expressed in the tailbud, presomitic mesoderm and developing somites. (C–F) *Wnt8a1* expressing cells are found in the tailbud at 2,5 days post fertilization (dpf) and 3,5 dpf and *Wnt8a2* is expressed in caudal hematopoietic tissue (arrows in D). Line in (F) marks the position of the transversal section shown in the inset. (G) After 4 dpf, *Wnt8a1* is expressed in the entire gut, oesophagus (not before 4 dpf (I)), swim- and gall bladder. The anatomy of these structures has been well described previously [40]. (I) The dissected gut system is shown for clarity. The inset shows *Wnt8a1* expression in the outflow tract of the dissected heart. (H) *Wnt8a2* transcripts are present in the otic vesicles. a, atrium; ca, caudal artery; cv, caudal vein; g, gall bladder; l, liver; no, notochord; nt, neural tube; oe, oesophagus; oft, outflow tract; ov, otic vesicle; psm, presomitic mesoderm; sb, swim bladder; som, somites; tb, tailbud; v, ventricle.

We find that medaka *Wnt8a1* is expressed in the axial part of the presomitic mesoderm in the tailbud (Figure 3A) [16] including cells of the most posterior notochord. Conversely, *Wnt8a2* expression is seen broadly throughout the posterior somatic mesoderm reaching into the forming somites (Figure 3B). While *Wnt8a1* remains expressed in a decreasing number of tailbud cells up to 3,5 dpf (Figure 3C,E) similar to zebrafish, *Wnt8a2* transcripts disappear in this part of the embryo. Instead, *Wnt8a2* starts to be expressed in cells of the caudal hematopoietic system at 2,5 days, where it is strongly expressed in the caudal vein from 3,5 dpf onwards (Figure 3D,F and inset) [39]. Moreover, expression is seen in the otic vesicles at 4 dpf (Figure 3H). On the other hand *Wnt8a1* transcripts appear in the entire gut by 3.5 dpf extending anteriorly into the oesophagus subsequently (Figure 3G,I). In addition, *Wnt8a1* is expressed in the outflow tract of the heart, the swim bladder and the gall bladder (Figure 3G,I and inset). These observations represent evolutionary diversification of gene expression within the teleost lineage and amongst vertebrate species in general. Unlike other model systems analyzed, we were not able to detect expression of zebrafish *Wnt8a* genes at stages after somitogenesis by in situ hybridization. Conversely, *Wnt8a* gene expression in cells of the developing hindbrain rhombomeres seen in various species including zebrafish appears not to be conserved in medaka [17], [28], [29], [33]. Moreover, *Wnt8a* expression in limbs and branchial arches of mouse and chick embryos is not seen in fish [36]. Intriguingly, expression in the gut, heart and otic vesicles appears to be conserved between medaka and mouse [36]–[38], while only medaka *Wnt8a* gene expression is found in the caudal hematopoietic tissue, gall- and swim bladder (Table 1).

In summary, the medaka *Wnt8a* paralogs have acquired very different sites of expression when compared to each other, but the sum resembles much of *Wnt8a* expression in animals with a single *Wnt8a* gene in their genome.

The early medaka *Wnt8a1* gene expression is similar to *Wnt8a* expression of other vertebrate species. However, the temporal-spatial dynamics of *Wnt8a2* expression appears to have diverged during teleost evolution. It is tempting to speculate that this may be due to differences on the transcriptional level such that for instance unlike zebrafish and Fugu *Wnt8a*, medaka may not have a bicistronic *Wnt8a* transcript [17], [18].

Our phylogenetic analysis shows that the zebrafish *Wnt8a* genes do not separate into the two *Wnt8a* gene clusters of other teleosts, which may explain their similar expression and semi-redundant function described in zebrafish [17]. Conversely, the separation of medaka *Wn8a* paralogous copies together with the divergence of their expression suggests that the two genes may have acquired different functions during evolution.

## Supporting Information

Figure S1 **Analysis of zebrafish** ***Wnt8a*** **gene expression.** (A,B) Dorsal views and (C,D,G,H) lateral views of zebrafish embryos labeled for *Wnt8a ORF1* and *Wnt8a ORF2* expression at stages indicated to the left. (A–D) Embryos at 80% epiboly and 22 hpf exhibit the described *Wnt8a* gene expression in cells of the blastoderm margin and the tail tip respectively; no labeling is detected using the *Wnt8a* sense probes (insets in A and B). (E–H) Low levels of blue color is ubiquitously distributed in the brain of 2 dpf and 4 dpf old embryos, which is however also seen using the sense probe (insets in E and F). (I) RT-PCR analysis to detect *Wnt8a* gene transcripts reveals low levels of *Wnt8a* gene expression in both heads and tails of zebrafish embryos. Therefore the color visible in the head is likely to be background. 80%, 80% epiboly; n.c., negative control; st., standard; tb, tailbud.
(TIF)
