# Correction: Divergent *Wnt8a* Gene Expression in Teleosts

**DOI:** 10.1371/journal.pone.0092758

**Published:** 2014-03-24

**Authors:** The PLOS ONE Staff

In Table 1, the columns are misaligned. This error was introduced during the production process in preparation for publication. Please see the correct Table 1 here:

**Table 1:** Sites of *Wnt8a* gene expression in medaka, zebrafish, Xenopus and mouse

	Medaka		Zebrafish		Xenopus	Mouse
	*Wnt8a1*	*Wnt8a2*	*Wnt8.1*	*Wnt8a.2*	*Wnt8a*	*Wnt8a*
maternal	+	-	+	n.r.	n.r.	-
blastoderm margin/embryonic ectoderm	+	+*	+	+	+	+
paraxial mesoderm	-	+	+	n.r.	+	+
tailbud	+	+	+	+	+	+
cht	-	+	-	-	n.r.	n.r.
gut/oesophagus	+	-	-	-	+**	+**
gall bladder	+	-	-	-	n.r.	n.r.
heart	+	-	-	-	n.r.	+
swim bladder	+	-	-	-	n.a.	n.a.
otic vesicles	-	+	-	-	n.r.	+
brain	-	-	-	+	+	+
fins/limbs, branchial arches, eye	-	-	-	-	n.r.	+
main references	(Yokoi et al., 2003)this report		(Kelly et al., 1995)(Lekven et al., 2001)(Narayanan et al., 2011)this report		(Smith and Harland, 1991)(Christian et al., 1991)(Christian et al., 1993)(In der Rieden et al., 2010)	(Bouillet et al., 1996)(Jaspard et al., 2000)(Summerhurst et al., 2008)(Martin et al., 2012)

*Wnt8.1* and *Wnt8.2* correspond to zebrafish *Wnt8a ORF1* and *Wnt8a ORF2*, respectively.

*, very transient; **, hindgut; +, expressed by in situ hybridisation; -, not expressed by in situ hybridisation. Abbreviations: cht, chaudal haematopoietic tissue; n.a., not applicable; n.r., not reported.
