# Supplementary figures and images for: Divergent Wnt8a Gene Expression in Teleosts
Source: PLoS One. 2014 Jan 20;9(1):e85303. doi: 10.1371/journal.pone.0085303 (PMC3896364; doi:10.1371/journal.pone.0085303)

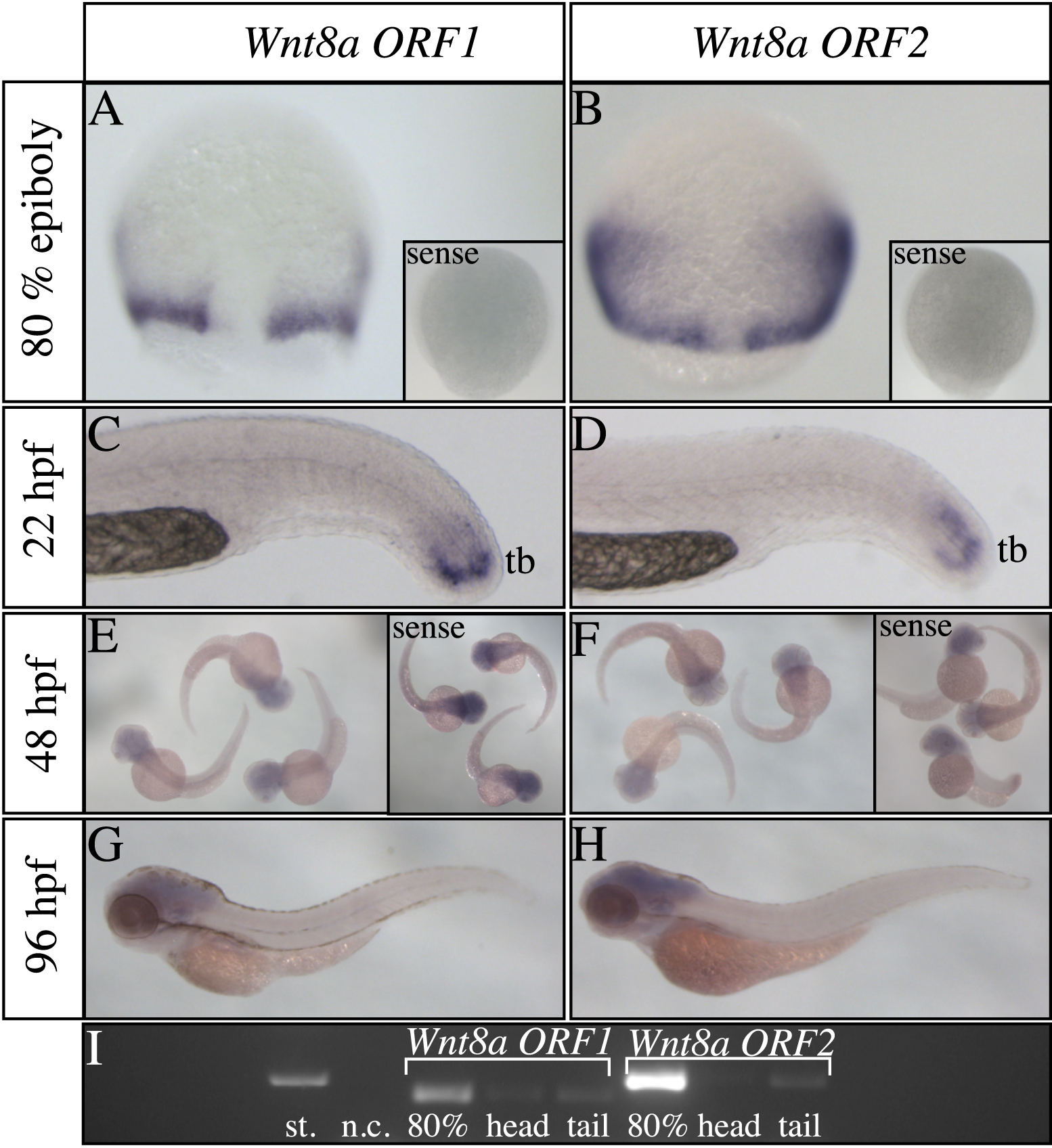

Supplement: Figure S1 — Analysis of zebrafish Wnt8a gene expression. (A,B) Dorsal views and (C,D,G,H) lateral views of zebrafish embryos labeled for Wnt8a ORF1 and Wnt8a ORF2 expression at stages indicated to the left. (A–D) Embryos at 80% epiboly and 22 hpf exhibit the described Wnt8a gene expression in cells of the blastoderm margin and the tail tip respectively; no labeling is detected using the Wnt8a sense probes (insets in A and B). (E–H) Low levels of blue color is ubiquitously distributed in the brain of 2 dpf and 4 dpf old embryos, which is however also seen using the sense probe (insets in E and F). (I) RT-PCR analysis to detect Wnt8a gene transcripts reveals low levels of Wnt8a gene expression in both heads and tails of zebrafish embryos. Therefore the color visible in the head is likely to be background. 80%, 80% epiboly; n.c., negative control; st., standard; tb, tailbud. (TIF) [file pone.0085303.s001.tif]
